# Fusion of apoptosis‐related protein Cytochrome c with anti‐HER‐2 single‐chain antibody targets the suppression of HER‐2+ breast cancer

**DOI:** 10.1111/jcmm.17001

**Published:** 2021-10-25

**Authors:** DanDan Lu, YiChen Guo, YunFeng Hu, Min Wang, Chen Li, Abhishek Gangrade, JiaHui Chen, ZiHui Zheng, Jun Guo

**Affiliations:** ^1^ School of Medicine & Holistic Integrative Medicine Nanjing University of Chinese Medicine Nanjing China; ^2^ Key Laboratory of Drug Target and Drug for Degenerative Disease Nanjing University of Chinese Medicine Nanjing China; ^3^ Department of Surgery and Biomedical Engineering University of Alabama at Birmingham (UAB) Birmingham Alabama USA

**Keywords:** breast cancer, HER‐2, immunoapoptotic molecules, scFv, targeted therapy

## Abstract

Cancer treatment has gradually developed from toxic chemotherapy to targeted therapy with fewer side effects. Approximately 30% of breast cancer patients overexpress human epidermal growth factor receptor 2 (HER‐2). Previous studies have successfully produced single‐chain antibodies (scFv) targeting HER‐2+ breast cancer; however, scFv have poor stability, easy aggregation and a shorter half‐life, which have no significant effect on targeting therapy. Moreover, scFv has been considered as a drug delivery platform that can kill target cells by effector molecules. However, the functional killing domains of immunotoxins are mainly derived from plant or bacterial toxins, which have a large molecular weight, low tissue permeability and severe side effects. To address these concerns, we designed several apoptotic immune molecules to replace exogenous toxins using endogenous apoptosis‐related protein DNA fragmentation factor 40 (DFF40) and tandem‐repeat Cytochrome c base on caspase‐3 responsive peptide (DEVD). Our results suggest that DFF40 or Cytc fusion scFv specifically targets HER‐2 overexpressing breast cancer cells (SK‐BR‐3 and BT‐474) rather than HER‐2 negative cells (MDA‐MB‐231 and MCF‐7). Following cellular internalization, apoptosis‐related proteins inhibited tumour activity by initiating endogenous apoptosis pathways, which significantly reduced immunogenicity and toxic side effects. Therefore, we suggest that immunoapoptotic molecules may become potential drugs for targeted immunotherapy of breast cancer.

## 1 INTRODUCTION

Breast cancer is known as the "pink killer" and represents the most common malignant tumour for women all over the world.^1^, ^2^, ^3^ In 1987, scientists discovered that the HER‐2 gene was amplified or overexpressed in approximately 20%–30% of breast cancers.^4^, ^5^ Excessive HER‐2 expression activates signal transduction pathways that lead to uncontrolled cell proliferation and tumour progression, and overexpressing patients also have correspondingly lower survival rates and faster relapse.^6^, ^7^, ^8^ Recently, antibody‐based immunotherapy for cancer has grown in popularity due to its specific targeting and remarkable efficacy. Clinically, the humanized monoclonal antibody, trastuzumab, has primarily been used to treat HER‐2 overexpressing metastatic breast cancer. Trastuzumab attaches itself to HER‐2 to block ligand binding and down‐regulate both angiogenic and metastatic genes.^9^, ^10^, ^11^ Despite the significant efficacy of trastuzumab, several patients continue to develop resistance and disease progression.^12^, ^13^, ^14^, ^15^

Single‐chain antibodies (scFv) are small molecule fragments formed by linking the heavy‐chain variable region (VH) and light‐chain variable region (VL) with a short (15 aa–20 aa), flexible peptide that has excellent targeting and reduced immunogenicity. The recombinant immunotoxin constructed with scFv as a vector‐induced cell death through the inhibition of toxin‐mediated translation; however, various toxins (e.g. pseudomonas exotoxin, staphylococcal enterotoxin and ribosome‐inactivating protein gelonin) have strong side effects, including gastrointestinal toxicity, liver toxicity, hypoalbuminemia and vascular leakage syndrome.^16^, ^17^, ^18^ To reduce these side effects, we incorporated endogenous apoptosis‐related proteins instead of plant or bacterial toxins with scFv, thereby inhibiting tumour progression by initiating apoptosis.

Apoptosis is an essential means by which the homeostasis of multicellular organisms is maintained and can selectively eliminate potentially harmful or infected cells.^19^, ^20^, ^21^ The accepted biological markers of apoptosis include chromatin concentration and DNA fragmentation,^22^, ^23^ which is primarily triggered by activation of the DNA fragmentation factor, DFF40. DFF40, known as caspase‐activated DNase (CAD), has been identified as a downstream molecule of caspase‐3. In addition, DFF40 and its natural inhibitor molecule, DFF45, typically constitute heterodimers. Previous studies indicate that upon DFF45 cleavage by activated caspase‐3, DFF40 was isolated and inhibition of its nuclease activity was lifted, which subsequently degrades double‐stranded DNA.^24^ DFF40 fusion expression and gonadotropin‐releasing hormone (GnRH) have been used as targeted therapy for adenocarcinoma.^25^ In addition, Cytochrome c (Cytc) also represents a key molecule involved in apoptosis.^26^ In the mitochondrial‐induced endogenous apoptotic pathway, after cells respond to apoptotic stimuli, the mitochondrial outer membrane is permeabilized, and Cytc is released into the cytoplasm to bind to apoptotic enzyme activating factor 1 (Apaf‐1). After recruiting pro‐caspase‐9, they form a giant Dalton complex: apoptotic bodies, which can promote caspase‐3 activation and induce apoptosis as described above.^27^, ^28^

Unlike necrosis,^29^ cells shrink following apoptosis, without the swelling and rupturing of organelles and plasma membranes. The cell membrane structure remains intact, and apoptotic cells eventually split into multiple apoptotic bodies wrapped in membranes, which can be quickly phagocytosed and cleared by phagocytes residing nearby.^30^ Such a process is not associated with many adverse effects (e.g. inflammatory reactions) and is a safer means of inhibiting tumour progression. This study aimed to construct immunoapoptotic molecules by fusing anti‐HER‐2 scFv as a binding domain with DFF40 or tandem‐repeat Cytc (3Cytc) and evaluate the anti‐tumour efficacy of these constructs.

## 2 MATERIALS AND METHODS

### 2.1 Cell lines and cultures

The human embryonic kidney cell line, HEK‐293T (ATCC®CRL‐11268), and human breast cancer cell lines, SK‐BR‐3 (ATCC®HTB‐30), BT‐474 (ATCC®HTB‐20), MDA‐MB‐231 (ATCC®HTB‐26), and MCF‐7 (ATCC®HTB‐22) were authenticated according to ATCC guidelines. All cells were cultured in DMEM medium (Gibco) supplemented with 10% FBS (Gibco) and a 1% mixture of penicillin and streptomycin (Invitrogen). The cells were routinely tested for mycoplasma contamination with negative results. All cells were grown at 37°C in a humidified atmosphere containing 5% CO_2_.

### 2.2 Generation of anti‐HER‐2 scFv and fusion scFvs

#### 2.2.1 Construction of recombinant expression plasmids

The gene encoding anti‐HER‐2 scFv was composed of the heavy‐chain variable region (VH) and the light‐chain variable region (VL) of trastuzumab, which were connected by a flexible peptide linker, (G_4_S)_3_. The scFv cDNA sequence was artificially synthesized and cloned into the prokaryotic expression vector, pET‐32a(+), thereby creating the recombinant expression plasmid termed pET‐32a(+)‐scFv. Either a DFF40 or Cytc coding sequence was inserted upstream of scFv through Bgl II and Nco I to construct the expression plasmid of the fusion protein, DFF40‐scFv or Cytc‐scFv. 3Cytc‐scFv was designed by simultaneously connecting three Cytc molecules through the caspase‐3 cleavage site, DEVD, and cloned into pET‐32a(+)‐scFv as described above. A list of the primers used for homologous recombination are listed in Table 1.

**TABLE 1:** Primers used for PCR

Insert	Primers
ScFV	F:5′‐CGACGACGACGACAAGGCCATGGCTGAAGTTCAGCTGGTTGAATCTGG‐3′ R:5′‐CGACGGAGCTCGAATTCGGATCCTTAGCGTTTAATTTCCACTTTGGTGC‐3′
DFF40	F:5′‐CCAGCACATGGACAGCCCAGATCTCATGCTCCAGAAGCCCAAGAGC‐3′ R:5′‐CAACCAGCTGAACTTCAGCCATGGTCTGGCGTTTCCGCACAGGCTG‐3′
Cytc	F:5′‐CCAGCACATGGACAGCCCAGATCTCATGGGTGATGTTGAGAAAGGC‐3′ R:5′‐CAACCAGCTGAACTTCAGCCATGGTCTCATTAGTAGCTTTTTTGAGATAAGC‐3′
3Cytc	

#### 2.2.2 Expression and purification of different protein constructs

All recombinant plasmids were transformed into *Escherichia coli* BL21(DE3) competent cells, selected by ampicillin. To obtain the maximum yield, the bacterial solution was added with isopropyl‐β‐d‐thiogalactopyranoside (IPTG) at a final concentration of 0.1, 0.2, 0.5 and 1.0 mM, and shaken for 12 h at 20, 25, 30 and 37°C. The cells were harvested, resuspended in the lysis buffer and sonicated for 20 min. After centrifugation, the supernatant was retained and filtered (0.45 μm) to be applied to a Ni‐NTA chromatography column (Qiagen). Bound proteins were eluted in a gradient with different concentrations of imidazole buffer and the fractions were dialysed against PBS overnight at 4°C. Each purified protein was concentrated with a 10–30 kDa MWCO ultrafiltration tube (Millipore) according to the molecular weight.

### 2.3 Analysis of protein characterization, expression, and binding activity

#### 2.3.1 Immunoblotting

Purified eluate proteins were denatured by boiling for 10 min and separated on a 10% SDS‐polyacrylamide gel and visualized by Coomassie blue staining. After transferred to a PVDF membrane, membrane was incubated with a mouse anti‐6His mAb (Yeasen) and a goat anti‐mouse IgG‐HRP (Proteintech) secondary antibody. Specific binding was detected with an enhanced chemiluminescence kit (Millipore).

#### 2.3.2 Immunofluorescence

Cells were incubated with 5 µg/ml of various protein constructs. After the non‐specific binding sites were blocked with 4% BSA, the cells were incubated with mouse anti‐6His mAb (1:100 dilution) followed by an incubation with a goat anti‐mouse IgG‐FITC (Proteintech) (1:200 dilution) antibody in the dark. The nuclei were stained with DAPI (Solarbio). Labelled coverslips were examined using a confocal laser scanning microscope (CLSM) (Leica).

### 2.4 Cytotoxicity assays: cell viability and cell proliferation activity

#### 2.4.1 CCK‐8 assay

Cells were treated with different protein constructs at 37°C for 48 h, and the concentration of the proteins varied from 0.01 to 100 nM. According to the instructions, CCK‐8 (Dojindo) reagent was added, incubated and the absorbance was immediately measured at 450 nm using a microplate reader (Bio‐Teck). Each experimental group was performed in triplicate.

#### 2.4.2 Crystal violet staining

Cells were subsequently fixed with 4% paraformaldehyde and reacted with a 1 ml crystal violet staining solution (Solarbio). Cell proliferation activity was evaluated by comparing the number of stained cells.

### 2.5 Apoptosis assays: morphological observations, apoptosis‐related protein analysis and flow cytometry

#### 2.5.1 Caspase‐3 activity assay

Cells stimulated as described above were digested and lysed. According to the manufacturer's instructions, the reaction solution and caspase‐3 cutting substrate‐Ac‐DEVD‐pNA (Jcbio) were added to establish the reaction system. The absorbance at 405 nm was measured using a microplate reader. Each experimental group was performed in triplicate.

#### 2.5.2 Level of Bcl‐2 and Bax expression

Cells treated with 100 nM of the different protein constructs were lysed with RIPA and the total protein concentration was confirmed using a BCA detection kit (Thermo). The denatured proteins were transferred to PVDF membranes as described above and incubated with antibodies specific to Bcl‐2 and Bax (CST) (1:1000 dilution). α‐tubulin (CST) (1:1000 dilution) was used as the internal reference.

#### 2.5.3 Annexin V‐FITC/PI double staining

A total of 5 × 10^5^ cells per well were seeded into six‐well plates and treated as described above. The cells were digested with EDTA‐free trypsin, collected and resuspended binding solution. Annexin V‐FITC and PI were added in sequence and incubated together with the cells in the dark. The processed samples were immediately detected using a BD FACS Calibur (BD Biosciences). The results were analysed with FlowJo software.

#### 2.5.4 Morphological changes

SK‐BR‐3 cells were seeded onto 9.6 cm^2^ coverslips overnight to achieve approximately 60% confluency and stimulated as described above. The nuclei were stained with Hoechst 33258 (Solarbio) and observed using an inverted fluorescence microscope (Leica). The nucleus of the apoptotic cells appeared bright white after shrinking.

In addition, the morphological changes of the SK‐BR‐3 cells were continuously recorded under a 20× microscope lens in a Live cell workstation (MD). The cell morphology at 0, 24 and 48 h under different treatment conditions was compared to assess the degree of apoptosis. The cells that appeared shrunken, blistered and divided into apoptotic bodies were considered to be apoptotic cells and quantified using Image J software.

### 2.6. In vivo nude mouse xenograft model

2.6

All animals were obtained from the Local Model Animal Research Center and housed under pathogen‐free conditions at 24°C. All procedures were performed in accordance with the guidelines established by the National Institutes of Health and approved by the Animal Ethics Committee of Nanjing University of Chinese Medicine.

Six‐week‐old female nude mice (BALB/c) were subcutaneously inoculated in the right armpit with 1 × 10^7^ SK‐BR‐3 cells mixed with matrigel at 4:1. When the tumours reached an average volume of 50 mm^3^, 20 mice were randomly divided into four groups and administered scFv, DFF40‐scFv, Cytc‐scFv or 3Cytc‐scFv. Ten doses of each protein construct were intraperitoneally administered at a dose of 10 mg/kg every three days. The tumour size was calculated every three days according to the following standard formula: *V* (mm^3^) = width^2^ (mm^2^) × length (mm)/2. At the experimental endpoint, all mice were euthanized, and the tumours were excised and weighed. The tumour tissues were fixed in paraformaldehyde, sliced into paraffin sections and analysed by a TUNEL Apoptosis Detection Kit (Jcbio). The DNA fragmentation of the apoptotic cells produced a 3′‐OH end, which appeared brown under an optical microscope.

### 2.7 Statistical analysis

Data analysis was conducted using the statistical program SPSS v.20.0 (IBM). All results were expressed as the mean ± SD. The statistical differences between groups were analysed by Student's *t* test and anova. A *p* value of <0.05 was considered significant.

## 3 RESULTS

### 3.1 Preparation and identification of different protein constructs

**FIGURE 1:**
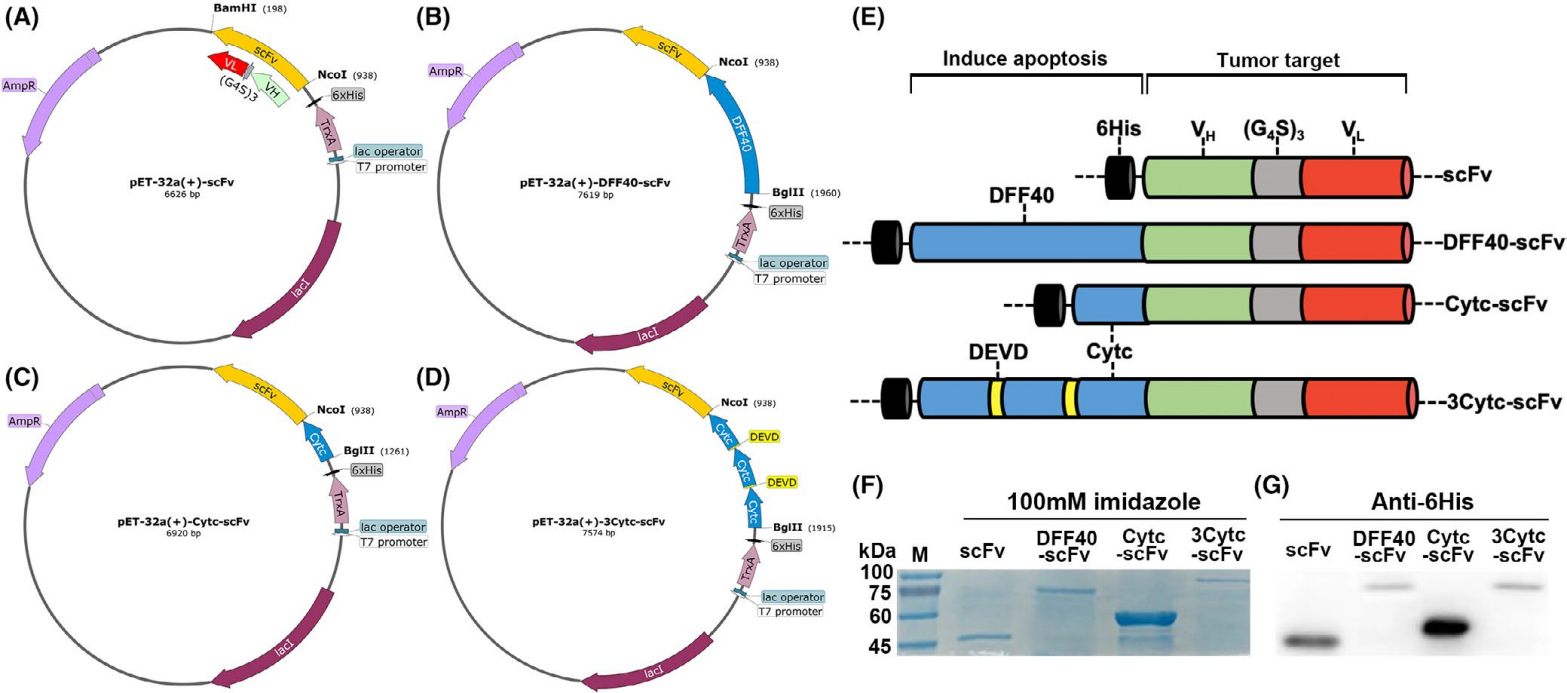
Genetic engineering technology: construction of recombinant proteins and verification of expression. (A–D) pET‐32a(+) recombinant plasmid maps were inserted with scFv, DFF40‐scFv, Cytc‐scFv and 3Cytc‐scFv gene sequences, respectively. All structural units were marked. (E) Two‐dimensional structural diagrams of the four protein constructs. It was mainly divided into two parts: (1) tumour‐targeting area and (2) apoptosis‐inducing area. (F) SDS‐PAGE electrophoresis results of four protein constructs purified by affinity chromatography. M denotes the Marker lane, followed by the 100 mM imidazole eluate of each protein construct. (G) Western blot results that further confirm the expression of all protein constructs. An anti‐6His mAb antibody was used

Artificially synthesized cDNA fragments were cloned into pET‐32a(+), a vector expressing soluble proteins via TrxAtag, to construct recombinant plasmids. The target genes consisted of scFv alone and three fusion constructs: DFF40‐scFv, Cytc‐scFv and 3Cytc‐scFv. All plasmid maps are shown in Figure 1A–D, with different colours representing different structural units. Figure 1E shows a two‐dimensional structure diagram clearly displayed for each component. All constructed recombinant plasmids were sequenced and demonstrated to harbour the correct coding sequence.

Target gene expression was controlled by the bacteriophage T7 promoter and lactose operon. The optimal induction conditions of *E*. *coli* were determined by the control variable method as 0.5 mM IPTG and a low temperature reaction for 12 h at 20°C. SDS‐PAGE confirmed that the purified proteins eluted from the Ni‐NTA column were primarily in the 100 mM imidazole eluent (Figure 1F). Based on the optical density analysis of the band, the purity of each protein was greater than 90%. After removing imidazole by dialysis, each protein construct was concentrated to 1–2 mg/ml in an ultrafiltration tube. Western blotting was performed to confirm protein expression via an anti‐6His antibody, and the specific combination was clearly visible at the corresponding position on the PVDF membrane. All recombinant plasmids successfully expressed target proteins following transformation into *E*. *coli* BL21 (DE3), with correct molecular weights of 46 kDa for scFv; 84 kDa for DFF40‐scFv; 58 kDa for Cytc‐scFv and 83 kDa for 3Cytc‐scFv (Figure 1G).

### 3.2 scFvs bind specifically to HER‐2+ breast cancer cells

**FIGURE 2:**
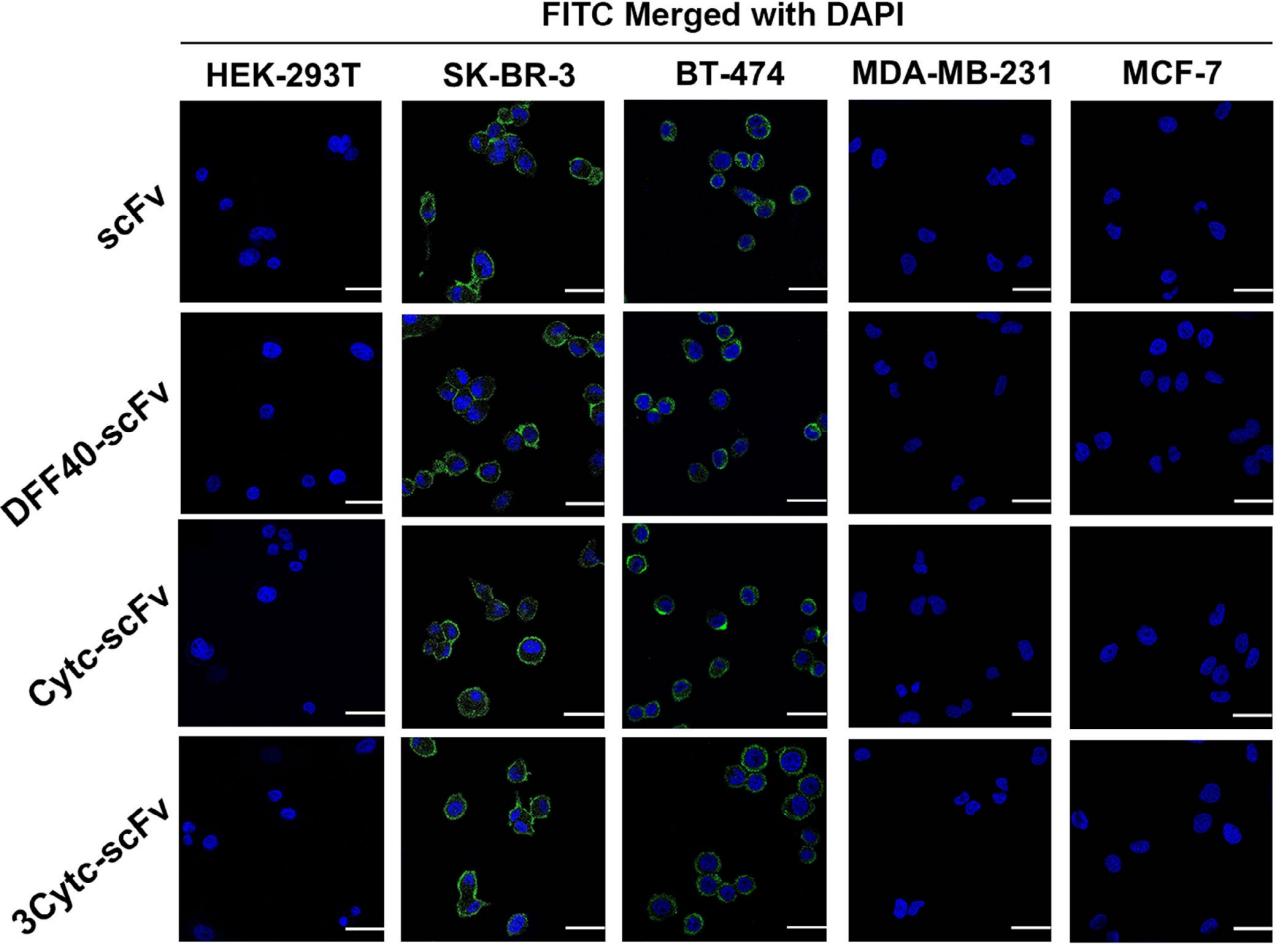
Targeting activity of the scFvs. Normal cells (HEK‐293T), high HER‐2 expressing cells (SK‐BR‐3 and BT‐474) and HER‐2 negative cells (MDA‐MB‐231 and MCF‐7) were incubated with scFv and three other fusion proteins, respectively at 37°C for 30 min. All treated cells were observed and imaged by confocal microscopy. Green denotes the target proteins labelled with a FITC fluorescent secondary antibody, and blue denotes the nucleus stained with DAPI. Scale bar = 25 µm

The targeting activity of scFv was investigated with an immunofluorescence analysis. Since breast cancer cells are divided into different subtypes according to the expression of the estrogen receptor, progesterone receptor, and HER‐2, we selected different types of cells to validate our protein constructs. Following an incubation with the purified proteins, both the single scFv and scFv fusion proteins were detected to bind strongly to HER‐2 high‐expressing breast cancer cells SK‐BR‐3 (ER‐/PR‐/HER‐2+) and BT‐474 (ER+/PR+/HER‐2+) cells (Figure 2). For normal cells, HEK‐293T and HER‐2 negative cells, MDA‐MB‐231 (ER‐/PR‐/HER‐2‐) cells and MCF‐7 (ER+/PR+/HER‐2‐) cells, which serving as controls, no green fluorescence was observed on the cytomembrane (Figure 2). These results indicate that the anti‐HER‐2 scFv prepared by the prokaryotic expression system specifically bound to the breast cancer cell surface antigen, HER‐2, and had equivalent level of targeting as monoclonal antibodies. Moreover, the binding activity was not impaired after coupling with DFF40 or Cytc.

### 3.3 Selective cytotoxicity of immunoapoptotic molecules to different tumour cells

**FIGURE 3:**
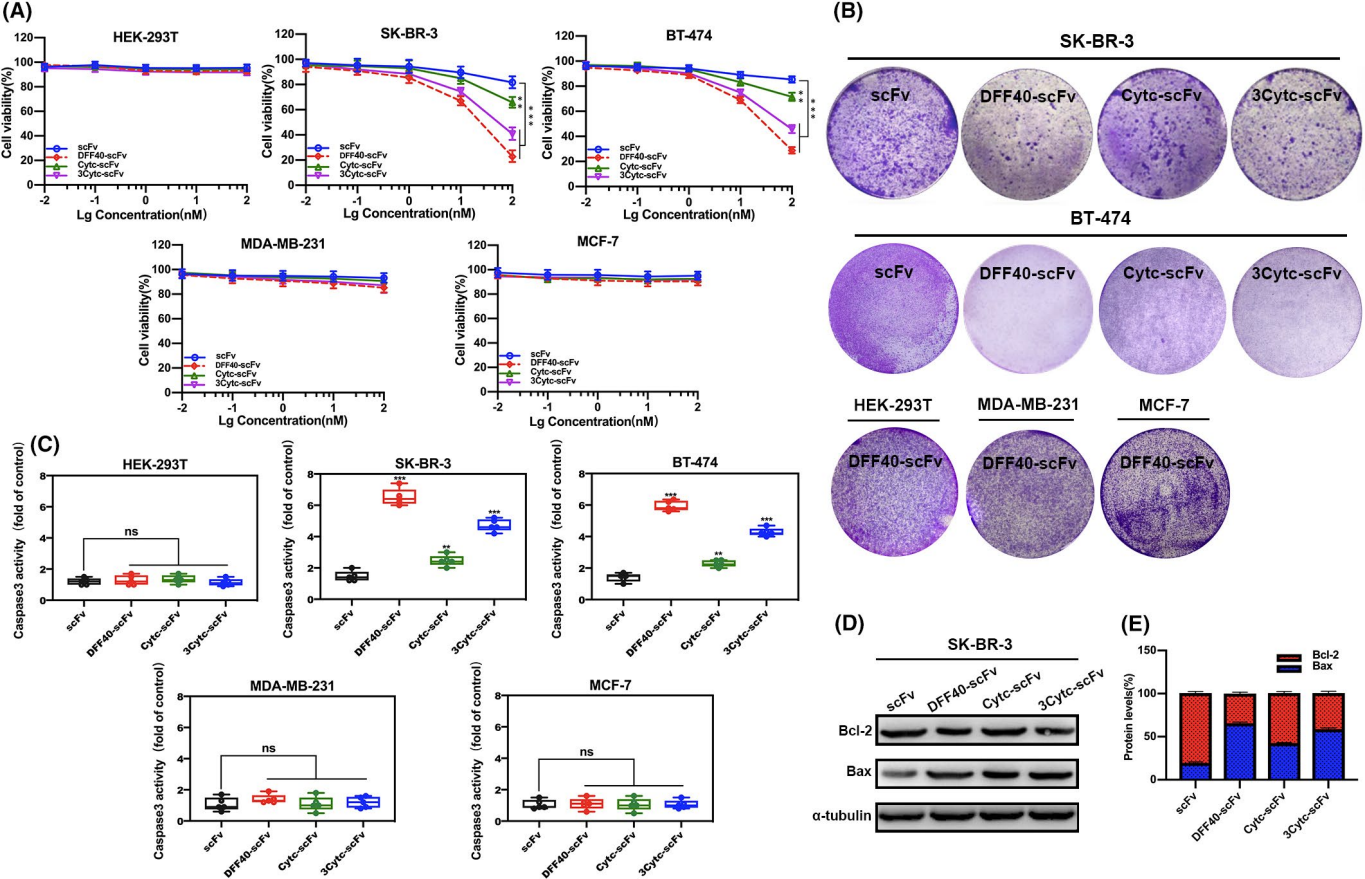
Cytotoxicity and apoptosis‐inducing activity of immunoapoptotic molecules in different cell lines. (A) Cytotoxicity data of normal cells (HEK‐293T), high HER‐2 expressing cells (SK‐BR‐3 and BT‐474) and HER‐2 negative cells (MDA‐MB‐231 and MCF‐7) treated with different concentrations of the protein constructs. (B) Crystal violet–stained images of cells stimulated with the different protein constructs. The proliferation activity was reflected by the number of cells. (C) The degree of caspase‐3 activation in the cells stimulated with the different protein constructs. (D) The level of expression of the apoptosis‐related proteins, Bcl‐2 and Bax, as detected by Western blot. α‐tubulin was used as an internal reference. (E) Statistical graph obtained by a quantitative analysis of protein bands in (D), reflecting the ratio of Bcl‐2/Bax. *n* ≥ 3 compared with the control group; **p < 0.01; ***p < 0.001; ns, not significant

Next, we closely monitored the viability of cultured cells exposed to each of the four respective constructs for 48 h. As shown in Figure 3A, the viability of the normal HEK‐293T cells was not affected. Although the growth of HER‐2+ breast cancer cells, SK‐BR‐3 and BT‐474, was effectively inhibited by fusion proteins, the single scFv was virtually non‐toxic. A concentration‐dependent reduction in vitality was observed. Under the same stimulation with 100 nM, DFF40‐scFv was the most toxic, exhibiting a nearly 80% decrease, followed by 3Cytc‐scFv, whereas Cytc‐scFv had low toxicity, in only approximately half of the 3Cytc‐scFv‐treated cells. In addition, little or no toxicity was detected in the HER‐2 negative cells, MDA‐MB‐231 and MCF‐7 (Figure 3A), which clearly indicated that the presence of higher levels of extracellular HER‐2 expression was required for the specific cytotoxicity of the fusion proteins.

Similarly, based on the crystal violet staining images presented in Figure 3B, the SK‐BR‐3 and BT‐474 cell colonies incubated with fusion proteins were significantly reduced compared to those incubated with single scFv. DFF40‐scFv exhibited the strongest inhibitory activity on cell proliferation, whereas 3Cytc‐scFv and Cytc‐scFv had a moderate and a weak effect, respectively, consistent with previous findings. Neither the growth of HEK‐293T nor MDA‐MB‐231 and MCF‐7 cells was affected by stimulation with DFF40‐scFv. Therefore, the presence of scFv was associated with the specific toxicity of immunoapoptotic molecules towards cells overexpressing HER‐2 without affecting the activity of normal cells.

### 3.4 DFF40 and Cytc induce target cell apoptosis

Previous studies have suggested that caspase‐3 is an executor of the caspase family and plays a key role in the apoptosis pathway.^27^, ^31^, ^32^ Here, the caspase‐3 specific cleavage sequence, DEVD, was coupled to the yellow group p‐nitroaniline, was released when the substrate was cleaved by activated caspase‐3, and could be detected at 405 nm. We measured the level of caspase‐3 activity to indirectly investigate whether scFv‐delivered DFF40 and Cytc could induce the apoptosis of target cells. As shown in Figure 3C, no caspase‐3 activation was observed in the HER‐293T cells, whereas the degree of caspase‐3 activation in SK‐BR‐3 cells stimulated with DFF40‐scFv, Cytc‐scFv and 3Cytc‐scFv was 6, 3 and 4.5 times higher than single scFv, respectively. The same trend was detected in BT‐474 cells. Similarly, no activation of caspase‐3 was detected in the HER‐2 negative cells, MDA‐MB‐231 and MCF‐7 (Figure 3C).

The immunoblotting results further confirmed the occurrence of apoptosis. The Bcl‐2 gene is one of the most important regulators of apoptosis‐related genes.^33^ Moreover, both Bcl‐2 and Bax are members of the Bcl‐2 family, in which the former resists apoptosis and then promotes apoptosis.^34^ Bcl‐2 can form a dimer with Bax. If the level of Bax expression is higher than that of Bcl‐2, it will promote apoptosis, otherwise apoptosis will be inhibited.^35^, ^36^ In SK‐BR‐3 cells, the level of Bcl‐2 in the cytoplasm was significantly reduced under the stimulation of DFF40‐scFv, and Bax was simultaneously increased. Moreover, the Bcl‐2/Bax ratio was confirmed to be reduced by quantification (Figure 3E). Similar to the trends observed in previous experiments, the Bcl‐2/Bax ratio under 3Cytc‐scFv stimulation also decreased, but to a lesser extent compared with that of DFF40‐scFv, whereas Cytc‐scFv did not have a substantial effect.

**FIGURE 4:**
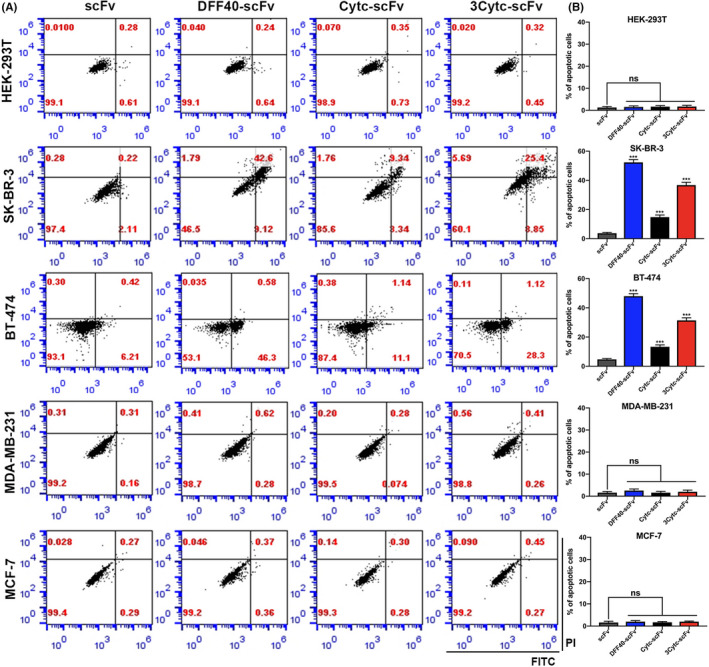
Flow cytometry detection of apoptosis induced by immunoapoptotic molecules. **(**A) The flow graphs of different cell lines treated with four kinds of proteins for 48 h. The abscissa is represented by Annexin V‐FITC and the ordinate is represented by PI. (B) Statistical graphs were obtained by performing a quantitative analysis of the apoptosis data in (A). *n* = 3, compared with the control group; ***p < 0.001; ns, not significant

Next, we assessed the level of apoptosis by flow cytometry. The abscissa and ordinate of the flow graph represented FITC and PI, respectively. The apoptotic cells primarily existed in the right two quadrants. In the HER‐2+ breast cancer cell lines, SK‐BR‐3 and BT‐474, the cells under scFv stimulation were largely Annexin V‐ and PI‐, indicative of normal cells. In contrast, the fusion proteins DFF40‐scFv, 3Cytc‐scFv and Cytc‐scFv caused significant apoptosis, which in turn decreased the apoptosis rate (Figure 4A,B). Similarly, none of the protein constructs induced apoptosis in the HEK‐293T control cells, and HER‐2‐ cells, MDA‐MB‐231 and MCF‐7 (Figure 4A,B). Together, these data indicate that single scFv could not induce apoptosis, and only exerted anti‐tumour activity when it was coupled with DFF40 or Cytc.

### 3.5 Significant morphological features of apoptosis

Apoptosis is associated with obvious morphological characteristics, including a reduced cell size, nuclear shrinkage, membrane bubbles, chromatin condensation, cytoskeletal disintegration and the formation of apoptotic bodies.^37^, ^38^ Although there are many methods that can be used to detect apoptosis, morphological observations are still reliable.

**FIGURE 5:**
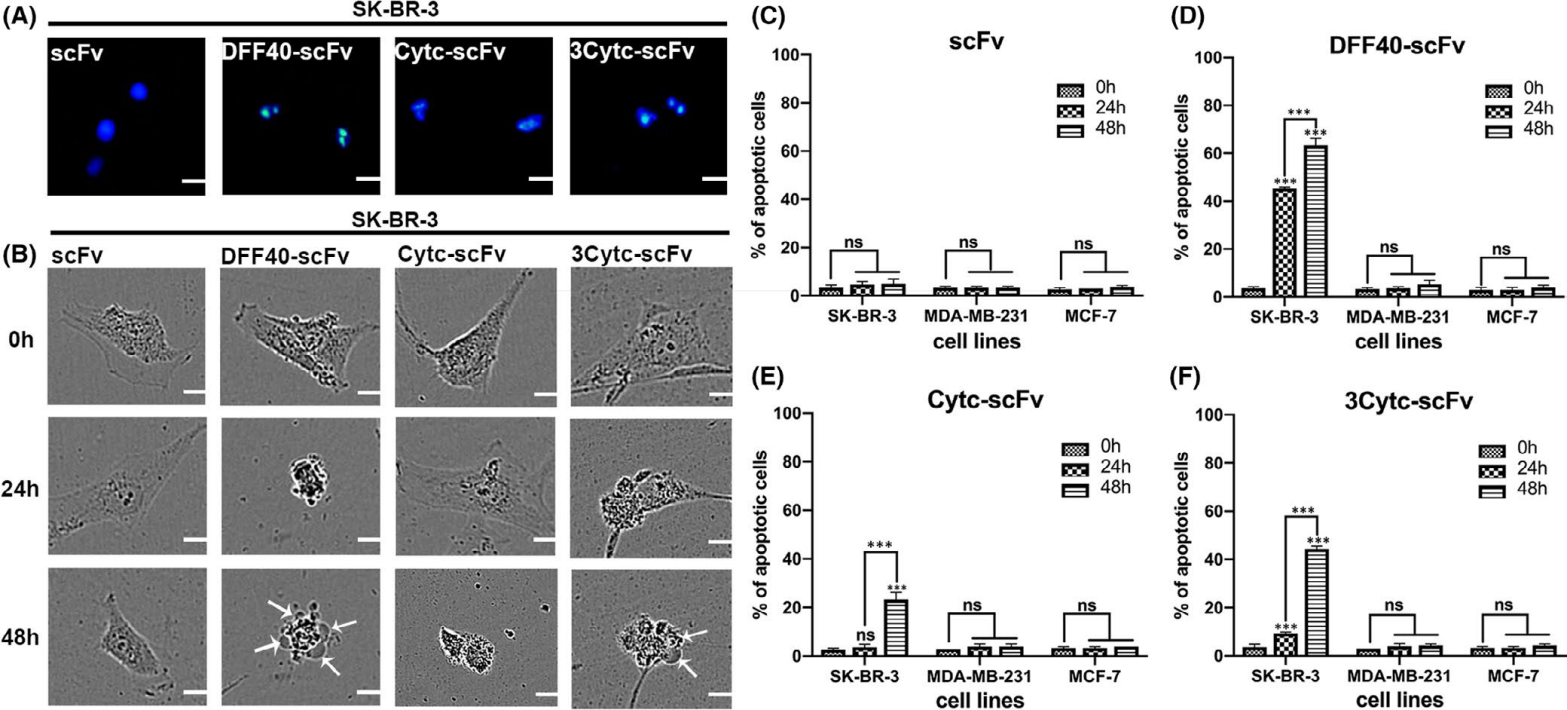
Morphology of the apoptotic cells. (A) The nuclei of SK‐BR‐3 cells incubated with different protein constructs showed varying degrees of shrinkage following Hoechst staining. (B) The morphological changes of SK‐BR‐3 cells at 0, 24 and 48 h under a long‐term imager. Bubbling positions were marked with white arrows. (C–F) Statistical graphs of the percentage of apoptosis in the SK‐BR‐3, MDA‐MB‐231 and MCF‐7 cell lines incubated with four protein constructs for 48 h. *n* = 3, compared with the control group; ***p < 0.001; ns, not significant

SK‐BR‐3 cells stimulated with different protein constructs were stained with Hoechst to label DNA and observed with a fluorescence microscope. As shown in Figure 5A, the cells under the influence of single scFv exhibited normal nuclei. In contrast, DFF40‐scFv was associated with ruptured nuclei and a colour change from blue to bright white, which represented the typical nuclear characteristics given that the chromatin shrunk during apoptosis. Consistent with the above findings, a similar phenomenon was observed in the 3Cytc‐scFv‐treated cells, whereas the nuclear changes in the Cytc‐scFv‐treated cells were slightly visible.

We also monitored the growth of cells in real time within 48 h of protein interaction using a live cell workstation. The morphology of SK‐BR‐3 cells treated with single scFv remained intact without apoptosis throughout the process. Following stimulation with DFF40‐scFv for 24 h, the cells contracted widely, and the volume was significantly decreased. Ultimately, bubbles appeared on the cell surface and fragmented apoptotic bodies formed at 48 h (Figure 5B). As expected, cells treated with 3Cytc‐scFv exhibited a dense cytoplasm at 24 h, with both blisters and apoptotic bodies also appearing at 48 h. The cells treated with Cytc‐scFv showed a normal morphology at 24 h, and contraction was observed until 48 h. Figure 5C–F present the apoptosis rate quantification. For the HER‐2 negative cells, MDA‐MB‐231 and MCF‐7, none of the four constructs induced apoptosis (images not shown). In summary, DFF40 or Cytc fusion scFv induced the apoptosis of target cells, which was associated with typical morphological changes. Although both DFF40‐scFv and 3Cytc‐scFv could definitively cause apoptosis, the former took effect at 24 h, whereas the latter was more obvious at 48 h.

### 3.6 Immunoapoptotic molecules inhibit breast tumours in vivo

**FIGURE 6:**
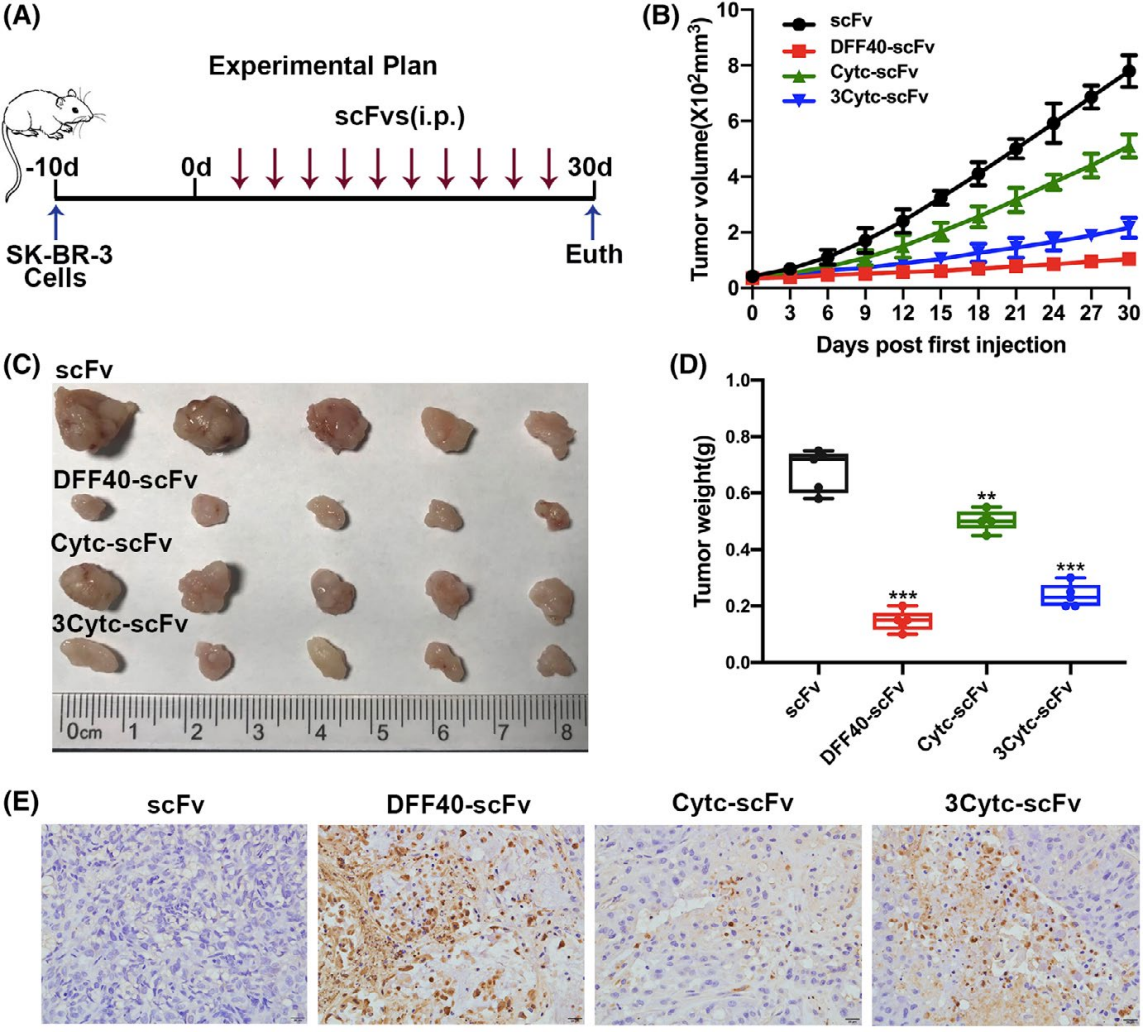
Anti‐tumour activity of immunoapoptotic molecules *in vivo*. (A) Schematic diagram showing the establishment of the tumour xenograft model in nude mice and the associated treatment plan. (B) Changes in the tumour volume were recorded every three days. (C) Tumour tissue was isolated at the end of the experiment. (D) Final quality of the tumour tissue. (E) Apoptosis rate in the tumour paraffin sections by TUNEL staining. Apoptotic cells appeared brown under a light microscope. *n* = 5, compared with the control group, **p < 0.01; ***p < 0.001

To confirm the clinical relevance of the suppression induced by the constructed immunoapoptotic molecules in HER‐2+ breast cancer, we subcutaneously injected SK‐BR‐3 cells into BALB/c nude mice to establish a breast cancer xenograft model. Proteins were administered intraperitoneally to treat the tumour‐bearing mice (Figure 6A).

Tumour progression in mice treated with fusion proteins, DFF40‐Cytc, 3Cytc‐scFv and Cytc‐scFv, was significantly suppressed compared with that of the scFv control group, and the anti‐tumour effects of the three were diminishing (Figure 6B–D). Images of TUNEL‐stained cells also showed different degrees of apoptosis in the tumour tissue between groups (Figure 6E), of which the DFF40‐scfv group exhibited the largest proportion of apoptotic cells. These data were consistent with the results of the in vitro experiments, indicating that the immunoapoptotic molecules DFF40‐scFv, Cytc‐scFv and 3Cytc‐scFv showed targeted suppression of HER‐2+ breast cancer cells in vivo. Importantly, DFF40 was associated with more potent anti‐tumour activity compared to that of Cytc, and the introduction of caspase‐3 cleavage site to tandem‐repeat Cytc enhanced such activity.

## 4 DISCUSSION

Cancer immunotherapy has attracted increased attention in recent years. Moreover, the combination of toxins and recombinant antibodies has made it possible to achieve tumour‐specific cell killing.^39^, ^40^, ^41^ Single‐chain antibodies represent novel genetically engineered antibodies that can often serve as drug delivery platforms due to their superior targeting activity. In preclinical studies, several single‐chain antibody recombinant immunotoxins have been found to target different cell surface antigens associated with tumour cells and function as effective anti‐cancer drugs.^42^, ^43^, ^44^ Although cellular damage is the primary method by which immunotoxins kill tumours, the adverse reactions caused by exogenous toxins in normal human tissues are the main factors that limit their clinical application. Therefore, the development of recombinant therapeutic molecules containing effector domains with low toxicity has become an important research topic.

In the present study, we constructed single‐chain antibody scFv based on the clinical drug, trastuzumab, which targets the HER‐2 antigen on the cytomembrane of breast cancer cells. Potential side effects continue to be an important topic throughout drug development and treatment, and trastuzumab is no exception. However, the cardiotoxicity of trastuzumab is controversial. One clinical study^45^ on trastuzumab cardiotoxicity retrospectively analysed cases of early cardiac dysfunction (CD) treated with trastuzumab and found that most patients had received previous anthracycline therapy and the observed CD in response to trastuzumab resembled the cardiomyopathy associated with previously described anthracycline cardiotoxicity. Although the doxorubicin‐induced cardiotoxicity generally appears to be dose‐dependent, the cardiotoxicity of trastuzumab does not appear to increase with higher cumulative doses. In another seven‐year follow‐up assessment^46^ of cardiac function in patients with HER2‐positive breast cancer, the result of the risk/benefit ratio of trastuzumab was found to be strongly in favour of trastuzumab therapy.

We developed DFF40 and Cytc to replace exogenous toxins and fused them with scFv to construct immunoapoptotic molecules. Such immunoapoptotic molecules deliver DFF40 or Cytc to target cells through scFv and initiate tumour destruction. Since DFF40 and Cytc are endogenous proteins, the non‐specific toxicity and immunogenicity induced by immunotoxins can be avoided. In addition, traditional anti‐HER‐2 therapeutic mAbs rely on the patient's immune system or block the HER‐2 signaling pathway to exert an tumour killing effect.^47^ Moreover, at least 70% of patients with HER‐2 positive breast cancer exhibit disease progression.^48^ Therefore, immunoapoptotic molecules can bypass the endogenous resistance mechanism, thereby greatly reducing the resistance to mAb‐based drugs.

In addition to DFF40‐scFv and Cytc‐scFv, we creatively constructed tandem‐repeat Cytochrome c (3Cytc‐scFv). It is widely known that the final step in the apoptosis cascade is the activation of the caspase‐3 executive protein, which inhibits DNA repair and initiates DNA degradation.^49^, ^50^ Moreover, caspase‐3 is known as a cysteine‐containing aspartate proteolytic enzyme, which specifically recognizes and cleaves the peptide bond on the aspartic acid residue of the target protein. Our results showed that the cleavage sequence of caspase‐3 was Asp‐Glu‐Val‐Asp (DEVD),^51^, ^52^ and 3Cytc‐scfv was formed by connecting three Cytc proteins in a series through DEVD. The results showed that fusion proteins can specifically bind to breast cancer cells overexpressing HER‐2 in vitro. Under the same treatment conditions, DFF40‐scFv was the most toxic, resulting in the greatest degree of apoptosis, whereas only a weak effect was observed for Cytc‐scFv. It was gratifying that even though the activity of 3Cytc‐scfv with the introduced caspase‐3 cleavage site was inferior to that of DFF40‐scFv, it was significantly better than Cytc‐scFv and could induce substantial apoptosis. Similar findings were obtained in vivo.

As a downstream molecule of the apoptosis cascade, the activation of DFF40 will lead to irreversible DNA damage, thereby quickly killing the cells.^24^ However, Cytc triggers a response process, which is first released from the mitochondria into the cytoplasm to form an apoptosome, which then activates apoptotic hydrolases.^26^, ^27^, ^28^ We considered that although DFF40‐scFv has a stronger tumour killing rate, it was also the most toxic and has a molecular weight that is too large to have a negative impact on the body, making it a less than ideal construct. Moreover, Cytc has a smaller molecular weight, fewer side effects, and displayed better pro‐apoptotic efficacy than DFF40. When 3Cytc‐scFv is internalized into the target cell and the fusion protein induces apoptosis, activated caspase‐3 can recognize the DEVD amino acid sequence between Cytc and cut it off. As the DEVD breaks, each 3Cytc‐scFv construct will simultaneously release three free Cytc molecules. This mechanism is thus a clever virtuous cycle that activates the apoptosis pathway and in turn perpetuates the effect. Importantly, 3Cytc‐scFv can only limit its anti‐tumour effects to apoptosis, thereby avoiding toxicity and other side effects, which are not available for DFF40‐scfv and many other types of immunoapoptotic molecules.

We can safely conclude that the single‐chain antibody recombinant immune molecules fused with apoptotic proteins constructed in this study effectively targeted the breast cancer surface receptor, HER‐2, and induced apoptosis both in vivo and in vitro. No toxic effects were detected in the HER‐2 negative cells. To the best of our knowledge, there are few reports investigating the combination of the apoptotic protein, DFF40, and a single‐chain antibody for the treatment of HER‐2 positive breast cancer. Importantly, 3Cytc‐scFv introduced with caspase‐3 restriction sites represents an important supplement to the strategy of treating tumours through endogenous apoptosis pathways. As an ideal immunoapoptotic molecule in this study, although 3Cytc‐scFv provides a more novel and effective tumour antibody treatment approach, outstanding problems remain to be solved prior to clinical application. First, we can improve the purification process to eliminate possible contaminants. Secondly, it is necessary to further optimize the dosing regimen in rodents and try to implement recombinant antibodies for the treatment of non‐human primates. Finally, according to the design concept for 3Cytc‐scFv, new small endogenous effector proteins can be developed for the combined use with Cytc. The above optimization and mechanistic research may lead to more effective anti‐tumour activity.

In summary, we combined single‐chain antibodies with apoptosis and prepared a variety of immunoapoptotic molecules by cloning technology. The 3Cytc‐scFv strategy of connecting multiple small pro‐apoptotic molecules in a series and releasing them through enzymatic cleavage demonstrated superior tumour‐specific killing activity with fewer side effects, which is in line with the concept of drug treatments with a high efficiency and low toxicity. Therefore, these findings support targeting tumour antibody pathways to induce apoptosis in the future of cancer treatment.

## CONFLICT OF INTEREST

All authors declare no financial competing interests. All authors declare no nonfinancial competing interests.

## AUTHOR CONTRIBUTIONS

**DanDan Lu:** Data curation (equal); Methodology (lead); Validation (equal); Writing‐original draft (lead); Writing‐review & editing (equal). **YiChen Guo:** Data curation (equal); Methodology (equal); Validation (equal); Writing‐original draft (equal); Writing‐review & editing (equal). **YunFeng Hu:** Data curation (supporting); Methodology (supporting); Validation (equal); Writing‐review & editing (supporting). **Min Wang:** Data curation (equal); Methodology (equal); Validation (equal). **Chen Li:** Methodology (equal); Validation (equal). **Abhishek Gangrade:** Validation (equal); Writing‐original draft (equal); Writing‐review & editing (equal). **JiaHui Chen:** Data curation (supporting); Methodology (supporting); Validation (equal). **ZiHui Zheng:** Validation (equal); Writing‐original draft (supporting); Writing‐review & editing (supporting). **Jun Guo:** Conceptualization (lead); Funding acquisition (lead); Project administration (lead); Resources (lead); Supervision (lead); Validation (equal); Writing‐review & editing (supporting).

## Data Availability

All data used to support the findings of this study are available from the corresponding authors upon request.
